# Studies on Shokyo, Kanzo, and Keihi in Kakkonto Medicine on Prostaglandin E_2_ Production in Lipopolysaccharide-Treated Human Gingival Fibroblasts

**DOI:** 10.1155/2016/9351787

**Published:** 2016-10-13

**Authors:** Toshiaki Ara, Norio Sogawa

**Affiliations:** Department of Pharmacology, Matsumoto Dental University, 1780 Gobara Hirooka, Shiojiri, Nagano 399-0781, Japan

## Abstract

We previously demonstrated that a kampo medicine, kakkonto, decreases lipopolysaccharide- (LPS-) induced prostaglandin E_2_ (PGE_2_) production by human gingival fibroblasts. In this study, we examined the herbs constituting kakkonto that exhibit this effect. Shokyo strongly and concentration dependently and kanzo and keihi moderately decreased LPS-induced PGE_2_ production. Shokyo did not alter cyclooxygenase-2 (COX-2) activity, cytosolic phospholipase A_2_ (cPLA_2_), annexin 1 and COX-2 expression, and LPS-induced extracellular signal-regulated kinase (ERK) phosphorylation. Kanzo inhibited COX-2 activity but increased annexin 1 and COX-2 expression and did not alter LPS-induced ERK phosphorylation. Keihi inhibited COX-2 activity and LPS-induced ERK phosphorylation but slightly increased COX-2 expression and did not alter cPLA_2_ and annexin 1 expression. These results suggest that the mechanism of shokyo is through the inhibition of cPLA_2_ activity, and that of kanzo and keihi is through the inhibition of COX-2 activity and indirect inhibition of cPLA_2_ activity. Therefore, it is possible that shokyo and kakkonto are clinically useful for the improvement of inflammatory responses.

## 1. Introduction

Periodontal disease is accompanied by inflammation of the gingiva and destruction of periodontal tissues, leading to alveolar bone loss in severe clinical cases. Prostaglandin E_2_ (PGE_2_), interleukin-6 (IL-6), and IL-8 are known to play important roles in inflammatory responses and tissue degradation. PGE_2_ has several functions in vasodilation, enhancement of vascular permeability and pain, and induction of osteoclastogenesis and is believed to play important roles in inflammatory responses and alveolar bone resorption in periodontal disease [1].

A kampo medicine, kakkonto (TJ-1), has been clinically used for various diseases such as common cold, coryza, initial stage of febrile diseases, and inflammatory diseases. There are several reports showing that kakkonto possesses antiallergic [2, 3] and antiviral [4–7] effects in animal and* in vitro* experimental models. Regarding anti-inflammatory effects, kakkonto has been reported to decrease PGE_2_ production in cultured rabbit astrocytes [8]. Recently, we reported that kakkonto suppresses lipopolysaccharide- (LPS-) induced PGE_2_ production by human gingival fibroblasts (HGFs) [9], as well as shosaikoto [10], hangeshashinto [11], and orento [12].

Kakkonto is constituted with seven herbs (kakkon, taiso, mao, kanzo, keihi, shakuyaku, and shokyo). Some herbs such as keihi and shokyo are known to possess anti-inflammatory effects and are clinically used to treat inflammatory diseases [13, 14]. However, which compositions in kakkonto primarily show this effect is unclear. In this study, to elucidate the effect of kakkonto on decreasing LPS-induced PGE_2_ production more precisely, we examined those herbs that constitute kakkonto and their mechanisms.

## 2. Materials and Methods

### 2.1. Reagents

The ingredients of kakkonto formula are shown in Table 1. Kakkonto was purchased from Tsumura & Co. Powders of six herbs (taiso, mao, kanzo, keihi, shakuyaku, and shokyo) were provided by Tsumura & Co. (Tokyo, Japan). Kakkon (*Puerariae Radix*) was purchased from Tsumura & Co. A hot water extract of kakkon was prepared as reported previously [15]. In brief, 10 g of kakkon was decocted for 1 h with 100 mL of water. The decoctions were mixed, concentrated, and lyophilized. The w/w yield of kakkon was 2.1%. Powders of herbs and kakkonto were suspended in Dulbecco's modified Eagle's medium (D-MEM, Sigma, St. Louis, MO) containing 10% heat-inactivated fetal calf serum, 100 U/mL penicillin, and 100 mg/mL streptomycin (culture medium) and were stored at 4°C overnight under shaking. Then, the suspension was centrifuged and the supernatant was filtrated through a 0.45 *μ*m pore membrane. Lipopolysaccharide (LPS) from* Porphyromonas gingivalis* 381 was provided by Professor Nobuhiro Hanada (School of Dental Medicine, Tsurumi University, Japan). Arachidonic acid, prostaglandin H_2_ (PGH_2_), NS-398 (cyclooxygenase-2 (COX-2) inhibitor), CAY10502 (cytosolic phospholipase A_2_ *α*- (cPLA_2_ *α*-) specific inhibitor), bromoenol lactone (calcium-independent PLA_2_- (iPLA_2_-) specific inhibitor), and thioetheramide-PC (secretory PLA_2_- (sPLA_2_-) specific inhibitor) were purchased from Cayman Chemical (Ann Arbor, MI). Other reagents were purchased from Nacalai Tesque (Kyoto, Japan).

### 2.2. Cells

HGFs were prepared as described previously [11]. In brief, HGFs were prepared from free gingiva during the extraction of an impacted tooth, with the informed consent of the subjects who consulted Matsumoto Dental University Hospital. The free gingival tissues were cut into pieces and seeded onto 24-well plates (AGC Techno Glass Co., Chiba, Japan). HGFs were maintained in the culture medium at 37°C in a humidified atmosphere of 5% CO_2_. For passage, HGFs were trypsinized, suspended, and plated into new cultures in a 1 : 3 dilution ratio. HGFs were used between the 10th and 15th passages in the assays. This study was approved by the Ethical Committee of Matsumoto Dental University (number 0063).

### 2.3. Measurement of Cell Viability

The numbers of cells were measured using WST-8 (Cell Counting Kit-8; Dojindo, Kumamoto, Japan) according to the manufacturer's instructions. In brief, the media were removed by aspiration and the cells were treated with 100 *μ*L of mixture of WST-8 with culture medium for 2 h at 37°C in CO_2_ incubator. Optical density was measured (measured wavelength at 450 nm and reference wavelength at 655 nm) using an iMark microplate reader (Bio-Rad, Hercules, CA), and the mean background value was subtracted from each value.

### 2.4. Measurement of PGE_2_

HGFs were seeded in 96-well plates (10,000 cells/well) and incubated in serum-containing medium at 37°C overnight. Then, the cells were treated with various concentrations of each herb or kakkonto in the absence or presence of LPS (10 ng/mL) for 24 h (200 *μ*L each well) in triplicate or quadruplicate for each sample. After collecting the culture supernatants, viable cell numbers were measured using WST-8 as described above.

The concentrations of PGE_2_ in the culture supernatants were measured by enzyme-linked immunosorbent assay (ELISA), according to the manufacturer's instructions (Cayman Chemical), and were adjusted by the number of viable cells. Data are represented as pg per 10,000 cells (mean ± SD).

### 2.5. Measurement of COX-2 and Prostaglandin E Synthase

COX-2 and prostaglandin E (PGE) synthase activities were evaluated as shown previously [16] with slight modifications. In brief, to estimate COX-2 activity, HGFs were treated with LPS and herb for 8 h, washed, and incubated in culture medium containing exogenous arachidonic acid (10 *μ*M). The concentrations of PGE_2_ in the supernatants were measured by ELISA. PGE synthase activity was determined after a 15 min incubation with exogenous PGH_2_ (10 nM), and the concentrations of PGE_2_ were measured. Data are represented as pg per 10,000 cells (mean ± SD).

### 2.6. Preparation of Cell Lysates

HGFs were cultured in 60 mm dishes and treated with combinations of LPS and herb for the indicated times. Then, cells were washed twice with Tris-buffered saline, transferred into microcentrifuge tubes, and centrifuged at 6,000 ×g for 5 min at 4°C. Supernatants were aspirated and the cells were lysed on ice in lysis buffer (50 mM Tris-HCl, pH 7.4, 1% Nonidet P-40, 0.25% sodium deoxycholate, 150 mM NaCl, 1 mM ethylene glycol bis(2-aminoethyl ether)tetraacetic acid (EGTA), 1 mM sodium orthovanadate, 10 mM sodium fluoride, 1 mM phenylmethylsulfonyl fluoride, 10 *μ*g/mL aprotinin, 5 *μ*g/mL leupeptin, and 1 *μ*g/mL pepstatin) for 30 min at 4°C. Then, the samples were centrifuged at 12,000 ×g for 15 min at 4°C, and the supernatants were collected. The protein concentration was measured using a BCA Protein Assay Reagent kit (Pierce Chemical Co., Rockford, IL).

### 2.7. Western Blotting

The samples (10 *μ*g of protein) were fractionated in a polyacrylamide gel under reducing conditions and transferred onto a polyvinylidene difluoride (PVDF) membrane (Hybond-P; GE Healthcare, Uppsala, Sweden). The membranes were blocked with 5% ovalbumin for 1 h at room temperature and incubated with primary antibody for an additional 1 h. The membranes were further incubated with horseradish peroxidase-conjugated secondary antibodies for 1 h at room temperature. Protein bands were visualized with an ECL kit (GE Healthcare).

Antibodies against COX-2 (sc-1745, 1 : 500 dilution), cPLA_2_ (sc-438, 1 : 200 dilution), annexin 1 (sc-11387, 1 : 1,000 dilution), and actin (sc-1616, 1 : 1,000 dilution), which detects a broad range of actin isoforms, were purchased from Santa Cruz Biotechnology (Santa Cruz, CA). Antibodies against extracellular signal-regulated kinase (ERK; p44/42 MAP kinase antibody, 1 : 1,000 dilution) and phosphorylated ERK (Phospho-p44/42 MAPK (Thr202/Tyr204) (E10) monoclonal antibody, 1 : 2,000 dilution) were purchased from Cell Signaling Technology (Danvers, MA). Horseradish peroxidase-conjugated anti-goat IgG (sc-2020, 1 : 20,000 dilution) was procured from Santa Cruz, and anti-rabbit IgG (1 : 20,000 dilution) and anti-mouse IgG (1 : 20,000 dilution) were purchased from DakoCytomation (Glostrup, Denmark).

### 2.8. Statistical Analysis

**Figure 1:**
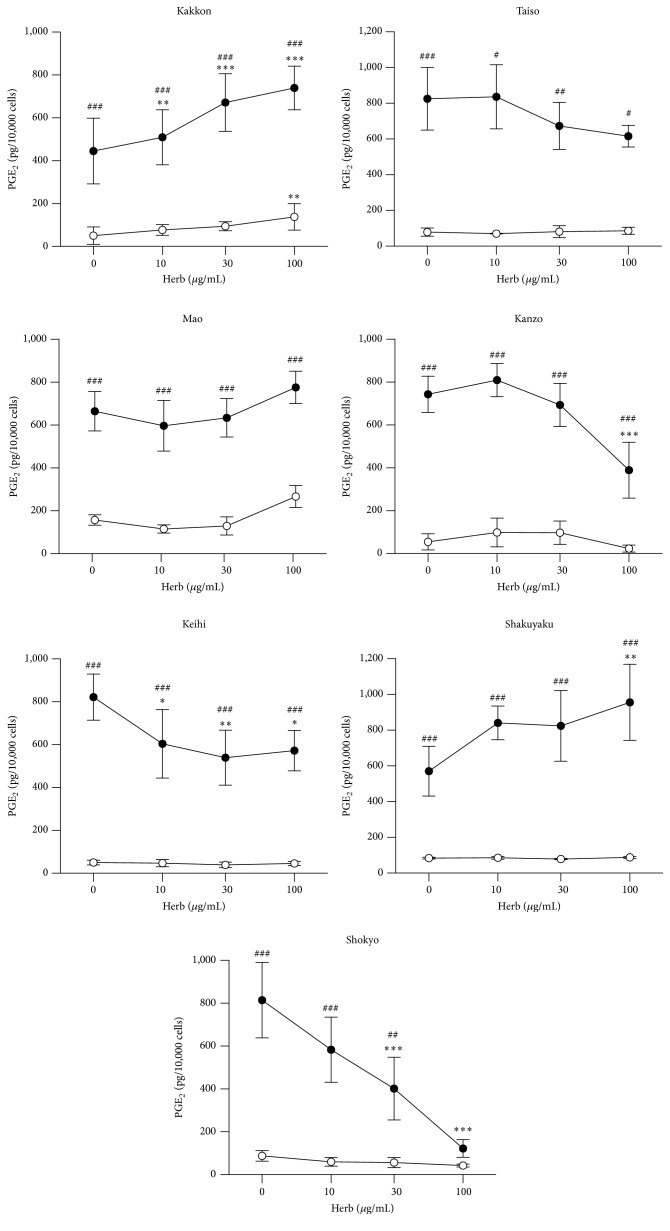
Effects of herbs on the production of PGE_2_. HGFs were treated with combinations of LPS (0 and 10 ng/mL) and herb (0, 10, 30, and 100 *μ*g/mL) for 24 h. Concentrations of PGE_2_ were measured by ELISA, adjusted by cell number, and expressed as pg per 10,000 cells (mean ± SD, *n* = 3). Open circles, treatment without LPS; closed circles, treatment with 10 ng/mL of LPS. ^*∗*^ *P* < 0.05, ^*∗∗*^ *P* < 0.01, and ^*∗∗∗*^ *P* < 0.001 (without herb versus with herb). ^#^ *P* < 0.05, ^##^ *P* < 0.01, and ^###^ *P* < 0.001 (without LPS versus with LPS). *P* values were calculated by pairwise comparisons and corrected with Holm's method (10 null hypotheses).

Differences between groups were evaluated using the two-tailed pairwise comparison test with a pooled variance, followed by correction with Holm's method (total of 10 null hypotheses; 3 null hypotheses without herb versus with herb in the absence of LPS, 3 null hypotheses without herb versus with herb in the presence of LPS, and 4 null hypotheses without LPS versus with LPS) (Figure 1). Differences between the control group and experimental groups were evaluated using two-tailed Dunnett's test (Figures 3 and 4).

All computations were performed with the statistical program R (http://www.r-project.org/). Dunnett's test was performed using the “glht” function in the “multcomp” package. Values with *P* < 0.05 were considered significantly different.

## 3. Results

### 3.1. Effect of Herbs on PGE_2_ Production

We examined whether the herbs affect LPS-induced PGE_2_ production by HGFs. The concentrations of PGE_2_ were adjusted according to viable cell number. When HGFs cells were treated with 10 ng/mL of LPS, HGFs cells produced large amounts of PGE_2_. Shokyo strongly and significantly decreased LPS-induced PGE_2_ production in a concentration-dependent manner (Figure 1). Kanzo and keihi moderately decreased LPS-induced PGE_2_ production (Figure 1). Taiso and mao had no effect on LPS-induced PGE_2_ production. Kakkon and shakuyaku increased LPS-induced PGE_2_ production (Figure 1). In the absence of LPS, kakkon increased PGE_2_ production, but kanzo decreased PGE_2_ production (Figure 1). Other herbs had no or little effect on PGE_2_ production. Therefore, we used three herbs (kanzo, keihi, and shokyo) in the following experiments.

**Figure 2:**
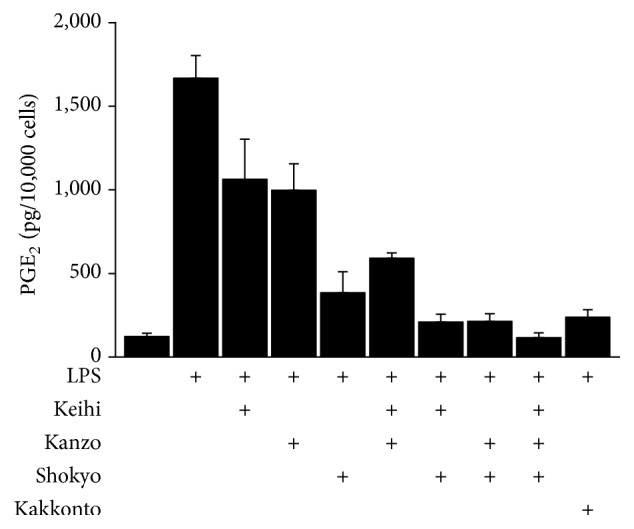
Effects of mixture of keihi, kanzo, and shokyo on the production of PGE_2_. HGFs were treated with combinations of LPS (10 ng/mL) and herbs (56 *μ*g/mL) or kakkonto (1 mg/mL) for 24 h. Concentrations of PGE_2_ were measured by ELISA, adjusted by cell number, and expressed as pg per 10,000 cells (mean ± SD, *n* = 4).

Next, we examined the synergistic effect of three herbs (shokyo, kanzo, and keihi) on PGE_2_ production and compared it with that of kakkonto. The concentrations of each herb (56 *μ*g/mL) were determined based on the ingredient of kakkonto formula (Table 1). The mixture of herbs further decreased PGE_2_ production. The combination of two herbs (shokyo + keihi and shokyo + kanzo) decreased PGE_2_ production to a similar level with 1 mg/mL of kakkonto. Moreover, the mixture of three herbs decreased PGE_2_ production more than kakkonto (Figure 2).

### 3.2. PLA_2_ Isoform Activities in HGFs

**Figure 3:**
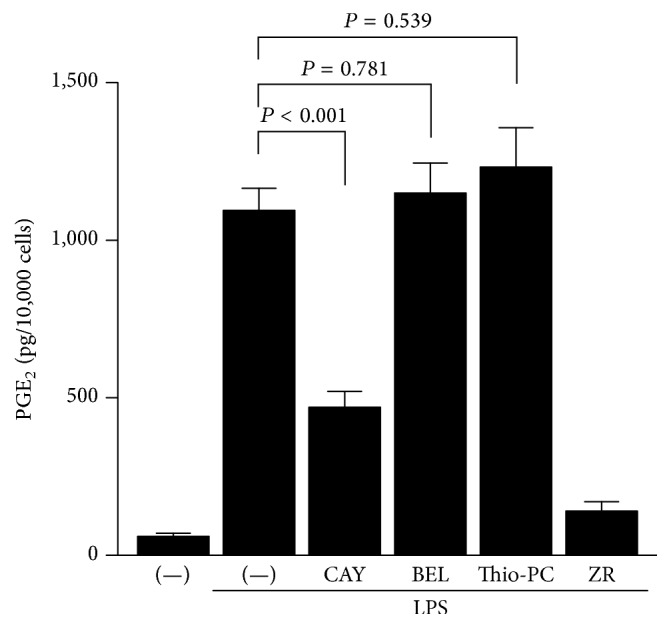
The contribution of PLA_2_ isoforms in HGFs. HGFs were treated with LPS (10 ng/mL) and PLA_2_ inhibitor for 24 h. Concentrations of PGE_2_ were measured by ELISA, adjusted by cell number, and expressed as pg per 10,000 cells (mean ± SD, *n* = 4). CAY10502, cPLA_2_ *α* inhibitor (100 nM); BEL (bromoenol lactone), iPLA_2_ inhibitor (20 *μ*M); Thio-PC (thioetheramide-PC), sPLA_2_ inhibitor (20 *μ*M); and ZR (shokyo, 100 *μ*g/mL) a positive control.

PLA_2_ is the most upstream enzyme in the arachidonic acid cascade and releases arachidonic acid from the plasma membrane. PLA_2_s form a superfamily and are classified into cytosolic PLA_2_ (cPLA_2_), calcium-independent PLA_2_ (iPLA_2_), secretory PLA_2_ (sPLA_2_), and others [13]. To elucidate which type of PLA_2_(s) contribute to arachidonic acid production in HGFs, we used selective PLA_2_ inhibitors. cPLA_2_ *α*-specific inhibitor CAY10502 significantly decreased LPS-induced PGE_2_ production by approximately half (Figure 3). However, both iPLA_2_-specific inhibitor BEL and sPLA_2_-specific inhibitor thioetheramide-PC did not alter LPS-induced PGE_2_ production (Figure 3). Therefore, we examined cPLA_2_ among these PLA_2_s in the following experiments.

### 3.3. Effect of Herbs on COX-2 and PGE Synthase Activities

**Figure 4:**
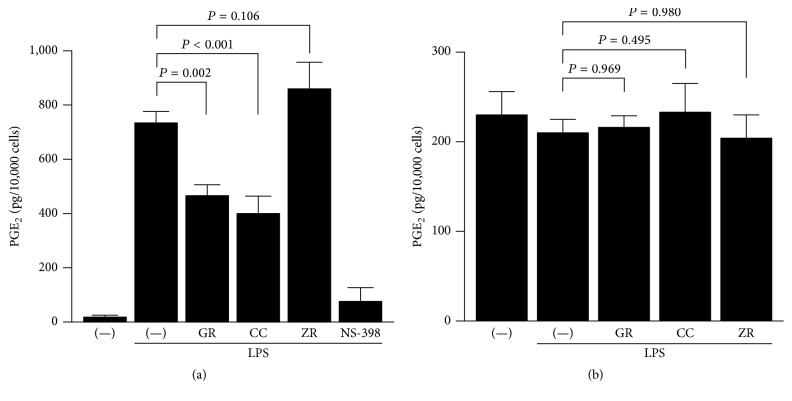
Effects of herbs on COX and PGE synthase activities. HGFs were treated with LPS (10 ng/mL) and herb (100 *μ*g/mL) for 8 h, washed, and then treated with (a) 10 *μ*M arachidonic acid or (b) 10 nM PGH_2_ for (a) 30 min or (b) 15 min. Concentrations of PGE_2_ were measured by ELISA, adjusted by cell number, and expressed as pg per 10,000 cells (mean ± SD, *n* = 3). *P* values by Dunnett's test are indicated. GR, kanzo; CC, keihi; ZR, shokyo; and NS-398, COX-2 inhibitor (20 *μ*M) as a positive control.

Then, we examined the mechanism by which kanzo, keihi, and shokyo decreased LPS-induced PGE_2_ production more directly. In order to bypass PLA_2_, we added exogenous arachidonic acid. Kanzo and keihi significantly decreased LPS-induced PGE_2_ production to approximately half, while shokyo slightly but not significantly increased PGE_2_ production (Figure 4(a)). NS-398, as a positive control, decreased LPS-induced PGE_2_ production.

The formation of PGE_2_ from arachidonic acid requires both COX and PGE synthase. To examine the effect of herbs on PGE synthase, we determined PGE_2_ formation from exogenous PGH_2_. However, all herbs had no effect on PGE_2_ formation from exogenous PGH_2_ (Figure 4(b)).

### 3.4. Effects of Herbs on Molecular Expression in the Arachidonic Acid Cascade

We examined whether herbs affect the expression of molecules in the arachidonic acid cascade. Kanzo increased cPLA_2_ expression, while keihi and shokyo showed no effect (Figure 6). Based on its molecular weight (approximately 90 kDa in human) [17, 18], this cPLA_2_ is believed to be cPLA_2_ *α* subtype.

Annexin 1, also named as lipocortin, is an anti-inflammatory mediator produced by glucocorticoids and inhibits cPLA_2_ activity [19, 20]. Kanzo increased annexin 1 expression, while keihi and shokyo showed no effect (Figure 6).

**Figure 5:**
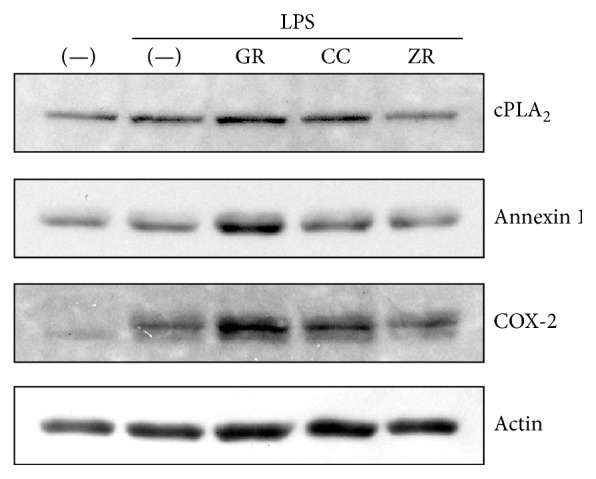
Effects of herbs on cPLA_2_, annexin 1, and COX-2 expressions. HGFs were treated with a combination of LPS (10 ng/mL) and herb (100 *μ*g/mL) for 8 h, and protein levels were examined by Western blotting. GR, kanzo; CC, keihi; and ZR, shokyo.

COX-2 was not detected in the absence of LPS and LPS-induced COX-2 expression in HGFs. Kanzo and keihi increased LPS-induced COX-2 expression. However, shokyo did not alter LPS-induced COX-2 expression (Figure 5).

### 3.5. Effects of Herbs on ERK Phosphorylation

**Figure 6:**
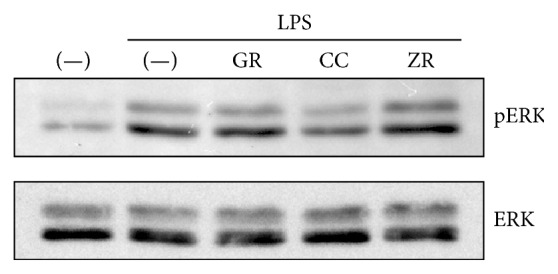
Effects of herbs on LPS-induced ERK phosphorylation. HGFs were untreated (0 h), treated with LPS (10 ng/mL), or treated with both LPS and herb (100 *μ*g/mL) for 30 min. Western blotting was performed using antiphosphorylated ERK or anti-ERK antibodies. pERK, phosphorylated ERK. Upper band indicates ERK1 (p44 MAPK) and lower band ERK2 (p42 MAPK). GR, kanzo; CC, keihi; and ZR, shokyo.

cPLA_2_ is reported to be directly phosphorylated at Ser505 by phosphorylated ERK, resulting in cPLA_2_ activation [21, 22]. Therefore, we examined whether herbs suppress LPS-induced ERK phosphorylation. Keihi suppressed LPS-induced ERK phosphorylation at 30 min, while kanzo and shokyo did not (Figure 6).

## 4. Discussion

In the present study, we examined the effect of herbs constituting kakkonto on LPS-induced PGE_2_ production by HGFs. Shokyo, kanzo, and keihi decreased LPS-induced PGE_2_ production in a concentration-dependent manner. In particular, shokyo showed the most marked effect. Previously, we examined the mechanisms of kakkonto [9] and shosaikoto [10] that contain shokyo and demonstrated that shosaikoto inhibited COX-2 activity and LPS-induced COX-2 expression and that kakkonto suppressed ERK phosphorylation. Based on our findings in the present study, shokyo is believed to play an important role in decreasing LPS-induced PGE_2_ production by HGFs in kakkonto and shosaikoto. In addition, the mixture of three herbs (shokyo, kanzo, and keihi) synergistically decreased PGE_2_ production (Figure 2). The effect of two herbs mixture including shokyo was comparable to that of kakkonto. Moreover, the effect of the three herbs mixture was stronger than that of kakkonto because mao and shakuyaku, which increase LPS-induced PGE_2_ productions (Figure 1), are not included. These results suggest that the combination of these herbs in kakkonto is sufficient to decrease PGE_2_ production.

Shokyo (*Zingiberis Rhizoma*) is the powdered rhizome of ginger (*Zingiber officinale* Roscoe). Several reports have shown that ginger has anti-inflammatory effects in humans, animal models, and* in vitro* models. Ginger has been widely used in diet and also as a treatment for rheumatoid arthritis, fever, emesis, nausea, and migraine headache [14]. Recently, a systematic review and meta-analysis reported that the extracts of Zingiberaceae including turmeric, ginger, Javanese ginger, and galangal are clinically effective as hypoanalgesic agents [23]. In an animal model, the aqueous extract of ginger significantly decreased serum PGE_2_ level by oral or intraperitoneal administration by the rat [24]. Moreover, crude hydroalcoholic extract of ginger reduced the serum level of PGE_2_ and improved tracheal hyperreactivity and lung inflammation induced by LPS in rat [25]. Ethanol extract of ginger reduced the tissue level of PGE_2_ and improved acetic acid-induced ulcerative colitis in the rat [26]. In* in vitro* model, gingerols and shogaols extracted from ginger are reported to decrease PGE_2_ production by several mechanisms. 10-Gingerol, 8, 10-shogaol [27], and 8-shogaol and 8-paradol [28] inhibit COX-2 activity. Moreover, gingerols, but not 6-shogaol, suppress COX-2 expression in LPS-treated human leukemic monocyte lymphoma U937 cells [29].

Our data showed that shokyo did not suppress COX-2 expression and that shokyo did not alter PGE_2_ production when arachidonic acid or PGH_2_ is added to bypass their upstream pathway. These data suggest that shokyo did not affect the downstream pathway of arachidonic acid, which includes COX-2 and PGE synthase. Therefore, shokyo is considered to inhibit PLA_2_, which is the upstream pathway of arachidonic acid. PLA_2_ hydrolyses the* sn*-2 ester bond of glycerophospholipids. Although PLA_2_s are classified into cPLA_2_, iPLA_2_, and sPLA_2_ [13], shokyo is suggested to act on cPLA_2_ because cPLA_2_ is the primary isoform in HGFs (Figure 3). Our data showed that shokyo only slightly decreased cPLA_2_ expression but did not alter annexin 1 expression, which suppresses PLA_2_ activity. Therefore, shokyo may primarily inhibit cPLA_2_ activity. Although we have no direct data to show that shokyo inhibits cPLA_2_ activity, this assumption is consistent with the fact that gingerols in ginger inhibit i/cPLA_2_ activities [30].

There are six molecules in cPLA_2_: cPLA_2_ *α*, cPLA_2_ *β*, cPLA_2_ *γ*, cPLA_2_ *δ*, cPLA_2_ *ϵ*, and cPLA_2_ *ζ* [31]. cPLA_2_ *α* was first identified and characterized by Ca^2+^-dependence and substrate preference for arachidonoyl phospholipids [32]. We detected cPLA_2_ at approximately 90 kDa as well as in human platelets and erythrocytes [17, 18], although the molecular weight of cPLA_2_ *α* protein on the basis of amino acid sequence is 85 kDa. Therefore, cPLA_2_ that we detected in HGFs is believed to be cPLA_2_ *α*. In contrast, cPLA_2_ *α*-specific inhibitor CAY10502 decreased LPS-induced PGE_2_ production to approximately half (Figure 3), suggesting that other cPLA_2_s such as cPLA_2_ *β* and cPLA_2_ *γ* may contribute to producing arachidonic acid, and shokyo may inhibit these cPLA_2_s. However, we could not detect cPLA_2_ *β* (114 kDa in humans), cPLA_2_ *γ* (61 kDa in humans), cPLA_2_ *ϵ* (100 kDa in murine), and cPLA_2_ *ζ* (96 kDa in murine) [13]. Although the molecular weight of cPLA_2_ *δ* from human/murine is 92-93 kDa [13], cPLA_2_ *δ* is distributed in the placenta [31]. These results suggest that there is no or very little contribution of cPLA_2_s other than cPLA_2_ *α* in HGFs; therefore, the remaining mechanisms remain to be elucidated.

As described above, our data that shokyo did not alter COX-2 activity and COX-2 expression are different from those of gingerols and shogaols. Although there is no obvious evidence, the reason may be the preparation method of shokyo. Gingerols and shogaols are extremely hydrophobic by their structures. Indeed, these compositions were extracted from hydrophobic phase in previous studies. However, the powders of herbs used in this study are prepared by decoction; therefore, hydrophilic compositions are likely to be extracted but hydrophobic compositions are unlikely to be extracted.

Kanzo (*Glycyrrhizae Radix*) is the powdered root or stolon of* Glycyrrhiza uralensis* Fischer. Kanzo is also known to have anti-inflammatory effects [33]. We demonstrated that kanzo decreased LPS-induced PGE_2_ production (Figure 1) and further demonstrated that kanzo increased annexin 1 expression (Figure 5), regardless of the increase of cPLA_2_ expression, suggesting that kanzo decreases LPS-induced PGE_2_ production by enhancement of annexin 1 expression and following inhibition of cPLA_2_ activity. However, the compositions that increase annexin 1 expression have not been reported. Moreover, we demonstrated that kanzo increased LPS-induced COX-2 expression (Figure 5). These findings are similar to those obtained using kampo medicines orento [12] and saireito [34], which contain kanzo. In contrast, kanzo decreased LPS-induced PGE_2_ production when arachidonic acid is added, while kanzo did not decrease when PGH_2_ was added (Figure 4). These results suggest that kanzo inhibits COX-2 activity but not PGE synthase. Indeed, kanzo inhibits COX-2 activity [35]. Therefore, kanzo inhibits arachidonic acid cascade in multiple points and cPLA_2_ and COX-2 activities. However, because the contribution of kanzo in kakkonto may be little, the ability of kanzo to decrease LPS-induced PGE_2_ production is weak. Kanzo contains the compositions such as glycyrrhizin, glycyrrhizic acid, liquiritin, and isoliquiritigenin. Nonetheless, the contributions of these compositions are unlikely in this study because they suppressed LPS-induced COX-2 expression [36–39]. Moreover, the compositions that inhibit COX-2 activity have not been reported. Therefore, other compositions may contribute to our findings.

Keihi (*Cinnamomi Cortex*) is the powdered bark of* Cinnamomum cassia*. Cinnamon has been widely used for the treatment of fever and inflammation [13]. Cinnamon improves nephritis, purulent dermatitis, and hypertension and enhances wound healing. Cinnamon extracts have been used for the improvement of or protection against common cold, diarrhea, and pain [13]. In a previous study, we demonstrated that ERK phosphorylation was suppressed by kakkonto [23] and orento [12], which also contains keihi. In this study, we demonstrated that this effect is responsible for keihi (Figure 6). Moreover, we demonstrated that keihi increased LPS-induced COX-2 expression (Figure 5) and that keihi decreased LPS-induced PGE_2_ production when arachidonic acid is added while keihi did not decrease when PGH_2_ was added. These results suggest that keihi inhibits COX-2 activity but not PGE synthase. Therefore, keihi inhibits arachidonic acid cascade in multiple points, cPLA_2_ activation, and COX-2 activity. However, the contribution of keihi in kakkonto may be little because the ability of keihi to decrease LPS-induced PGE_2_ production is weak. Keihi contains the compositions such as cinnamic aldehyde, cinnamic alcohol, cinnamic acid, and coumarin. Cinnamic aldehyde, but not others, suppressed LPS-induced COX-2 expression and decreased PGE_2_ production by RAW264.7 cells [40, 41]. Moreover, cinnamic aldehyde suppressed carrageenan-induced COX-2 expression and improved footpad edema in mouse [40]. However, the contribution of cinnamic aldehyde is unlikely in this study.

Aspirin-induced asthma (AIA) occurs after ingestion of acid nonsteroidal anti-inflammatory drugs (NSAIDs) such as aspirin and indomethacin [42, 43]. It is believed that AIA is caused by leukotorienes (LTs), in which contract bronchus are increased by acid NSAIDs [42, 43]. Similarly, acid NSAIDs are known to exacerbate a usual asthma. In this study, we speculate that shokyo inhibits cPLA_2_ activity. Therefore, the production of LTs is believed to be decreased because shokyo blocks arachidonic acid cascade at cPLA_2_ level. In this case, shokyo may be safely used for patients with asthma, including AIA, instead of conventional anti-inflammatory drugs. Moreover, oral administration of ginger protects against aspirin-induced gastric ulcers in rats [44]. Therefore, shokyo is possible to be available as an anti-inflammatory drug instead of NSAIDs.

## 5. Conclusion

We demonstrated that shokyo strongly and kanzo and keihi moderately decreased LPS-induced PGE_2_ production. Moreover, shokyo may inhibit cPLA_2_ activity and kanzo and keihi inhibit COX-2 activity directly and cPLA_2_ activity indirectly. These results suggest that shokyo, and kakkonto, is clinically useful for the improvement of inflammatory responses in periodontal disease and other diseases.

## Figures and Tables

**Table 1:** The ingredient of kakkonto formula.

Japanese name	Latin name	Amount (g)	Amount (g/g of product)^*∗*^
Kakkon	*Puerariae Radix*	4.0	0.111
Taiso	*Zizyphi fructus*	3.0	0.083
Mao	*Ephedrae Herba*	3.0	0.083
Kanzo	*Glycyrrhizae Radix*	2.0	0.056
Keihi	*Cinnamomi Cortex*	2.0	0.056
Shyakuyaku	*Paeoniae Radix*	2.0	0.056
Shokyo	*Zingiberis Rhizoma*	2.0	0.056

Total		18.0	0.500

^*∗*^7.5 g of kakkonto product contains 3.75 g of a dried extract of the mixed crude drugs.
